# Book Reviews

**DOI:** 10.1556/JBA.2.2013.4.10

**Published:** 2013-04-10

**Authors:** Csilla Ágoston

**Affiliations:** Doctoral School of Psychology Department of Clinical Psychology and Addiction Eötvös Loránd University Budapest, Hungary


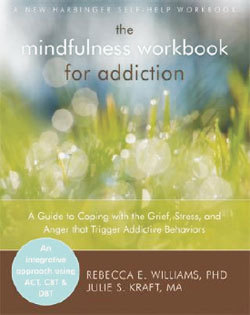


Rebecca E. Williams, PhD, the first author of *The mindfulness workbook for addiction* received her master's degree from Harvard University and her PhD from the University of California, Santa Barbara. She is a clinical psychologist specialized in recovery from mental illness and addictions and also the coauthor of *Couple therapy for alcoholism* (Wakefield, Williams, Yost & Patterson, 1996). She is currently the director of the Veterans Affairs San Diego Health-care System's Wellness and Vocational Enrichment Clinic. She represents not only the practical, but also the scientific field of psychology, as she is an associate clinical professor in the Department of Psychiatry at the University of California, San Diego and an adjunct faculty member at the University of San Diego.

The other author, Julie S. Kraft, MA, received her master's degree in marriage and family therapy from the University of San Diego's School of Leadership and Education Sciences. She works at the Veterans Affairs San Diego Health-care System as a counselor, and provides psychotherapy to individuals, couples, families and groups in community settings. In her current position at Sharp HealthCare she treats clients struggling with both addiction and mental health concerns. She is also familiar with acceptance & commitment therapy (ACT), cognitive behavioral therapy/REBT, contemplative psychotherapy, dialectical behavioral therapy, emotionally focused couples therapy, emotionally focused therapy, interpersonal psychotherapy, mindfulness-based approaches, object relations, person-centered/Rogerian therapy and narrative therapy.

*The mindfulness workbook for addiction* is a self-help book, not an academic one. There are several approaches of treating addictive behaviors, however, this book presents an altogether new and holistic direction. The book offers useful practices for the addicted individuals. Furthermore, it is also profitable for professionals, mainly due to the fact that it compounds the elements of several therapeutic interventions, therefore offers an extensive approach for addiction treatment. The way in which the authors guide the reader is logically coherent and encompasses a real therapeutic depth.

At the very beginning of the book we can read about the story of a fictional couple. The husband, Tony Gomez, has alcohol problems and his wife, Carmen, is a compulsive buyer. After getting to know the story of the Gomez family, we can keep track of their increasing self-awareness and recovery while their life becomes more and more meaningful. The couple – and possibly most readers – has a negative attitude towards some of the chapters or exercises. The authors, however, offer coping strategies for these problems throughout the book to facilitate compliance.

After the introduction of the family comes the guideline to aid the use of the book and to establish the reader's goals. The workbook consists of three main parts and ten chapters. The chapters will be introduced one after the other for a deeper understanding.

The chapters of the first part start relatively far from the topic of addictions. The issues of emotions, thoughts and behaviors are discussed thoroughly, so that the reader can acquire several mindfulness practices. In Chapter 1 *(Emotions)* Williams and Kraft draw attention to the incorrect schemes ingrained in childhood and they suggest some exercises – for example one including a sort of cognitive restructuring – to break them down. They reveal how can we feint our emotions – often by the addictive behavior – and then they present some exercises which can help the individual to identify emotions emerging in various situations. This chapter also includes a useful chart that contains a systematized and sophisticated variety of emotions.

The authors rely on Beck's theory (1991), namely that thoughts evoke emotions which contribute to the birth of further thoughts. In Chapter 2 *(Thoughts)* there is a strong emphasis on repeat-offender thoughts. Brief descriptions of the cognitive behavioral therapy techniques are also common in this chapter, and there are a lot of examples and exercises as well. This section provides a variety of options and good practices for restructuring maladaptive thought patterns. The authors describe five types of disturbing thoughts, so that the reader can easily recognize them in his or her own way of thinking.

After recognizing, understanding and rectifying emotions and thoughts Chapter 3 *(Behaviors)* focuses on behavior and behavioral change. The authors describe exercises connected mainly to two therapeutic interventions, the acceptance and commitment therapy (ACT) and the dialectical behavior therapy (DBT). At this point, the authors describe the concept of DBT and encourage the reader to carry out some therapeutic exercises which may aid to replace the maladaptive behavioral patterns with adaptive ones. In the sequel the chapter starts by focusing on values, which can be a new basis of the rearrangement of behavior. The readers are guided by some ACT exercises in order to explore their own values to discover what really matters in their lives. The authors point out that the elements of our value system can conflict with each other; therefore they show us how to build up systematic decisions related to the current behavior in tough situations.

Chapter 4 *(Mindfulness),* which terminates the first part of the book, targets the development of mindfulness skills which were established and extensively studied by Kabat-Zinn (2003). Fundamental practices are described, which enable the individuals to thoroughly observe their minds at the present time without judgment, to understand the nature of stress, to be present through experiencing space, sounds and objects as well as to practice extreme acceptance. These exercises help addicted individuals to gain a greater balance in their emotions, thoughts and behavior, thereby raising their problem-solving skills to a higher level.

The second part – which contains the topic of losses, addictions and the junction of these two – is less extent, but it captures the roots of the problem. Out of the possible reasons of the development of addictive behaviors Williams and Kraft underline the importance of losses and highlight the individual's recent and previous losses in Chapter 5 *(Loss)*. The authors draw attention to the fact that losses may have numerous shapes. “Most of the time, when you hear the word ‘loss’, you think of death, but the truth is that in the course of your life, there are many significant losses. Here's a good way to start defining loss: think of a loss as any time when you've said good-bye to something. You could say good-bye to a relationship, the house you grew up in, a job, a school, a town. It may be that you said good-bye to a dream when you realized you would never play professional baseball or be a concert-level pianist. You may have been forced to say good-bye to your innocence at a very young age; someone or something may have stolen your childhood from you through trauma.” Readers are encouraged to become aware of the variety of possible losses by a long list. Furthermore, they are also encouraged to sort the most important losses and to reveal to what extent they are elaborated and how they affect the reader's current life.

By this point, the addicted individual's resistance and denial of addiction would have been dissolved and they would presumably have engaged in the process. Based on these assumptions, Chapter 6 *(Addiction)* may be able to discuss the topic of addictions more easily. After delineating the definition and some examples the individuals can choose from a list which substance or substances they have been using and then they can carry out a self-reported diagnosis. The same steps are repeated with the behavioral addictions as well. Setting up the timeline of an addictive behavior can also be very useful, informative and even eye-opening for the addicted individual. This timeline appears also in Chapter 7 *(Connecting addiction and loss),* which presents the link-up of the individual's addictive behaviors and experienced losses. The delineated loss-addiction cycle helps to understand how the experience of a significant loss can lead to addictive behaviors and how the extant addiction can provoke more losses (e.g. the partner leaves the addicted person because of the addiction).

The chapters in the third part are aimed at healing through conscious grief, rebuilding relationships and relapse prevention. In Chapter 8 *(Mindful grieving)* one can find several other advanced mindfulness practices, which promote mindful grief and empower the individual to be more flexible and resistant instead of suppressing emotions and problems.

Chapter 9 *(Relationships)* is an especially well-positioned and thoroughly discussed part of the book. It primarily aims rebuilding relationships. Up to this point the individuals could practice their new skills in a secure setting, but now they will have to try them out in a social context. This attempt can be very stressful because of the unpredictable and possibly negative reactions. However, the individual will have acquired coping skills by this time which can help them through the potential difficulties. The exercises can support the recognition and elimination of unhealthy affiliations beyond the restoration of the old and ruined relationships. The individual can also learn how to build new, healthy relationships and maintain those by using some helpful communication skills.

The last chapter *(Recovery, relapse prevention, and beyond)* exceeds the problem of addictions and focuses on the preparation of a healthier life. The field of nutrition, sleep, exercise, work and entertainment is displayed and all of them contribute to the permanent improvement of life quality. To reach further successes the authors provide a list of helpful books and Web sites for the reader.

Great merit of the book is that each chapter gives the reader a positive confirmation of the results achieved so far. Williams and Kraft make a constant effort to maintain the cooperation of the addicted individuals. The reinforcements are placed in such parts of the book where the reader confronts with ponderous contents or self-discovery challenges, such as reviewing losses (Chapter 6), connecting losses with addictions (Chapter 7) or learning the way of mindful grieving (Chapter 8).

Similar to the language of the book which is full of metaphors, the process described by the book can be pictured as a spiral-shaped path leading to the top of a high mountain. The road is long, but due to the sinuous path it is not too steep and the more we advance, the more we find beautiful landscapes by looking around along the way. The present book offers this gradual but firmly upward path to be followed. *The mindfulness workbook for addictions* is particularly useful for addicted people but provides a helpful guide for professionals as well.
