# Supplementary material for: Book Reviews
Source: J Behav Addict. 2013 Apr 10;2(4):253–7. doi: 10.1556/JBA.2.2013.4.10 (PMC4154575; doi:10.1556/JBA.2.2013.4.10)
Supplement: Related texts

# Sub article

**Authors:** Csilla Ágoston

**Affiliations:** Doctoral School of Psychology Department of Clinical Psychology and Addiction Eötvös Loránd University Budapest, Hungary



Treating addictions is a great challenge for several reasons. The lack of treatment resources and consensus about therapies, the high treatment demand and the several associated problems imply remarkable issues to be solved by providers and researchers.

Systematization of recent efficient, moreover cost-effective treatments and introduction of their adaptability for different kinds of addictions are the greatest virtues of the book.

Steven C. Hayes, PhD, the first editor of *Mindfulness & acceptance for addictive behaviors,* obtained his PhD at West Virginia University in the doctoral program in clinical psychology.

As a well-known researcher of contextual behavioral science, he is a Foundation Professor at the Department of Psychology at the University of Nevada, Reno and also founder of acceptance and commitment therapy (ACT), which is substantially described in the present book. Professor Hayes has written 35 books including books about ACT's application for different disorders, self-help books and has numerous scientific articles as well; he had the merit of being the 30th “highest impact” psychologist in the world in 1992. Dr. Hayes has been President of several organizations such as the American Association of Applied and Preventive Psychology and earned a lot of awards, inter alia, for his applied scientific work.

Editor Michael E. Levin, MA, is a doctoral candidate in clinical psychology at the University of Nevada, Reno and is currently working on the evaluation of a transdiagnostic web-based ACT program. Moreover, within the confines of several funded grants, he has been focusing on the prevention of suicidality and mental health issues. Levin's main publications target addictive behaviors, experiential avoidance and mindfulness-based interventions.

At the beginning of the book the authors introduce the headway of state of the art interventions and offer a broad approach under the comprehensive label of *contextual cognitive behavioral therapies* which “tend to emphasize contextual and experiential change strategies in addition to more direct and didactic ones, and seek the construction of broad, flexible and effective repertoires over eliminative approaches to narrowly defines problems or syndromes” (p. 6). Different therapeutic methods belong to this conception, which are thoroughly described in the first part of the book, but the core common features are listed in the introduction. Despite the similarities, there are some theoretical differences such as treatment goals – e.g. whether reaching absolute abstinence is required or not.

The book is divided into two major parts. The chapters of the first part describe five therapeutic methods, each aimed at treating addictive behavior. The second part deals with some special clinical issues and pay heed to the treatment opportunities of some behavioral addictions, such as problem gambling, binge eating and pornography addiction.

The first therapeutic method described in the book by Wilson, Flynn and Kurz is acceptance and commitment therapy in Chapter 1. An important goal of this treatment is reaching psychological flexibility. According to the authors, there are six key components which the intervention is based on: attention focused on the present moment, healthy ways of relating to the self-as-content, self-as-process and self-as-context, resolving experimental avoidance (acceptance), defusion, attention to values and engaging with committed action. The assessment and intervention is demonstrated by a case study. An exercise from ACT's *Therapy guide (Practicing our way to stillness)* is also delineated for the better understanding of the six core processes. As a registered, evidence-based therapy, its elements can be integrated into various treatment settings.

In Chapter 2 dialectical behavior therapy (DBT) for substance use disorders is presented by Dimeff and Sayrs. DBT has a standard form, which was developed primarily for patients with borderline personality disorder (BPD) and which is “considered the gold-standard treatment of BPD” (p. 69). Because of the given antecedents, DBT for substance use disorders (DBT-SUD) is highly appropriate for patients who have both BPD and SUD; the co-morbidity of these two disorders is considered to be quite high. Since BPD-SUD patients usually have various severe problems, DBT lines up a hierarchy for the problems to be solved – e.g. life-threatening problems have greater priority than decreasing behavior which interferes with therapy. The functions of the DBT-SUD program are also described, and the path to clear mind – which is an ultimate goal in the therapy – is detailed. “(…) in clear mind, you work hard at getting clean and really appreciate the success of being clean, but you do not forget that getting clean isn't the endpoint” (p. 80). DBT-SUD is based on the philosophy of dialectics associated with the theories of Marx and Hegel; the therapist encourage the patient to reach a synthesis in a given topic wherein equilibrium of acceptance and change has an important role. This type of intervention has a dialectical approach referring to abstinence as well: striving for harm reduction and for absolute abstinence prevail alternately. Several studies provide evidence for the effectiveness of DBT superior to treatment as usual and some other interventions in different aspects.

The mindfulness-based relapse prevention program (MBRP) described by Bowen, Witkiewitz and Chawla in Chapter 3 contains the most mindfulness elements compared to the other treatments presented in this book. The main goal of MBRP is reducing the probability and severity of relapses for individuals in early abstinence focusing on three key aspects of addictions – namely nonacceptance, negative and positive reinforcement. Witkiewitz, Marlatt and Walker (2005) incorporated the elements of cognitive behavioral therapy and mindfulness meditation and finally developed the eight-session, structured protocol of MBRP which is detailed session by session in this chapter. Potential mechanisms and feasibility of MBRP has been studied and the results verify the effectiveness of this treatment.

In Chapter 4 Spada, Caselli and Wells describe metacognitive therapy. This approach highlights the so-called self-regulatory executive function (S-REF) model and a thinking style called cognitive attentional syndrome (CAS) which is activated by positive and negative metacognitive beliefs and which contributes to problem drinking. The authors review the role of metacognitive knowledge and metacognitive monitoring in the three phases of a drinking episode: pre-alcohol use, alcohol use and post-alcohol use phase. Beliefs linked to uncontrolled alcohol use, suppression of alcohol-related thoughts and extended thinking are delineated and the reader can get to know the elements of the metacognitive therapy as well. Testified forms of metacognitive therapy have already been established for different disorders – e.g. depression, generalized anxiety disorder, OCD, PTSD and social anxiety disorder. The presented model has been developed especially for problem drinking. Nevertheless, because of the novelty of this form of treatment further evidence is needed to demonstrate its efficiency.

In Chapter 5 Wagner, Ingersoll and Rollnick introduce the technique of motivational interviewing (MI) which is somewhat an exception to the presented treatments. According to the authors it can be seen as a cousin of contextual cognitive behavior therapies, because they have similarities – such as focusing on making discrete changes – and differences as well, like the role of the practitioner who elicits both problems and solutions from clients in the MI model, while lists problems from clients, and provides expert solutions in the CBT context. MI can be further differentiated from the other methods by its direct approach to change contrary to other methods which have a greater emphasis on the underlying psychological patterns. This method has an over 20-year-old past, therefore, its effectiveness has been reviewed extensively. Motivational interviewing has small to medium effect sizes for substance use but the effect is larger in case of engagement in substance abuse treatment, therefore, it is the most effective treatment of addictions except for CBT. MI's perspective, core communication skills and process are also described in this chapter.

Jennifer H. R. Sayrs unfolds the potential of mindfulness practices for professionals in the beginning of the second part of the book. This topic has mainly been ignored despite of the benefits of the practices which affect not only the providers’ quality of life but also that of the therapy. Sayrs summarizes the main mindfulness skills and introduces examples for each practice. Based on colleagues’ and own experiences the author reveals the positive influence of mindfulness in several situations such as dealing with clients’ hostility and critiques, being more present with the clients and avoiding the term “should”. Special issues like mindfulness practice for providers with a history of substance use are discussed. This chapter also provides a guide to the construction of one's own practice and answers the probable concerns for the compatibility of mindfulness with personal views. Another query is whether mindfulness conflicts with the 12-step program; however, there are great endeavors to combine these treatments (e.g. Jacobs-Stewart, 2010).

Stigmatizing caused by the common negative attitude toward addicted individuals is an important interfering factor in the treatment of addictions. Luoma and Kohlenberg undertake the review of the development and handling of stigma, self-stigma and shame in Chapter 7. They discuss the context which primes shame and self-stigma and introduce an evaluated ACT group protocol session by session which aims at overstepping both of them.

Detoxification can be a particularly hard part of the treatment for the patients with substance use disorder, but, as presented by Stott and Masuda in Chapter 8, the adaptation of mindfulness practices can be fruitful in this field as well. It is crucial for the patient to accept the existence of withdrawal symptoms and urges instead of fighting and suppressing them. The authors framed an acceptance- and mindfulness-based protocol in virtue of existing ACT manuals for the three phases of detoxification: pre-dose-reduction, dose-reduction and post-dose-reduction phase and follow-up. Empirical evidence supports the effectiveness of ACT-based intervention – almost twice as many patients in the ACT treatment group (36.7%) had an opiate-negative drug screen compared to the drug counseling group (19.2%).

As effective alternatives of pharmacotherapy Bricker and Wyszynsky review motivational interviewing and acceptance and commitment therapy for smoking cessation in Chapter 9. There is a description of therapeutic relationship, language and values in both therapies, illustrated with lifelike examples. Three forms – face-to-face, telephone and web-based – of intervention and their measured or probable efficacy for smoking cessation are discussed as well.

In Chapter 10 the possible application of mindfulness for problem gambling is brought forward by Tony Toneatto. Mindfulness-based interventions can possibly complement cognitive behavioral therapy and enhance its efficacy in the treatment of problem gambling. The author emphasizes the rigid dysfunctional cognitive beliefs of gamblers more than impulsive features and explains why concentration and insight aspects of mindfulness meditation are considered to be important components of the treatment. Toneatto draws attention to the fact that there are indirect evidences for the positive effects of mindfulness for problem gambling so far, e.g. higher trait mindfulness predicts more successful judgment on the Iowa Gambling Task. Nonetheless, mindfulness is a promising approach in this field.

Treatment opportunities of binge eating and chronic food overconsumption are reviewed by Jason Lillis in Chapter 11. The reader can get to know the features which drug abuse and overeating have in common including the neuro-biological background. Lillis lists four contextual CBT intervention techniques which aim at treating binge eating, food overconsumption and related problems. All interventions have components which can be highly effective for some aspects of the problem. In brief, ACT's significant asset is managing self-stigma and shame, DBT focuses on emotion dysregulation, motivational interviewing effectively helps to resolve ambivalence about change and mindfulness-based cognitive therapy includes some disorder-specific techniques such as the mindful eating exercise. Related studies, where available, are discussed and special issues are explicated as well.

In Chapter 12 Crosby and Twohig highlight the contextual approach (a special ACT protocol in particular) to the treatment of pornography addiction. The authors emphasize the functional nature of the problem, which can help to define the latent reasons of viewing including negative thoughts and emotions. Some case series and a randomized clinical trial with high effectiveness and special challenges, such as religious beliefs are introduced.

First and last, *Mindfulness & acceptance for addictive behaviors* is more than a great summary of new therapeutic trends on the addiction treatment field for professionals. It proves evidence for the applicability of cost-effective mindfulness-based programs which can be used independently from and integrated to other therapies. The authors assign the rightful place of the so-called contextual behavioral therapies for substance use and behavioral addictions. This book is an indispensable resource for mental health providers, addictions professionals and researchers, interested in cutting-edge treatments for addictive behaviors.

## References

[RS1] Jacobs-Stewart T (2010). Mindfulness and the 12 Steps: Living recovery in the present moment.

[RS2] Witkiewitz K, Marlatt G. A, Walker D. D (2005). Mind-fuless-based relapse prevention for alcohol use disorders. Journal of Cognitive Psychotherapy.
